# Endometrial expression of ERRβ and ERRγ: prognostic significance and clinical correlations in severe endometriosis

**DOI:** 10.3389/fendo.2024.1489097

**Published:** 2024-11-29

**Authors:** Zhenna Wang, Sang Guo, Yi Xie, Yao Tong, Wei Qi, Zhenhong Wang

**Affiliations:** Department of Obstetrics and Gynecology, Fujian Maternity and Child Health Hospital, College of Clinical Medicine for Obstetrics & Gynecology and Pediatrics, Fujian Medical University, Fuzhou, China

**Keywords:** severe endometriosis, estrogen receptor-related receptor-beta, estrogen receptor-related receptor-gamma, infertility, pregnancy

## Abstract

**Background:**

Endometriosis (EMs) results in approximately 50% of reproductive-age women facing infertility. Currently, no precise model is available to predict successful postoperative pregnancy.

**Methods:**

This study involved 81 patients with severe EMs (stages III and IV) and 38 controls with benign gynecological conditions, matched by age and BMI, diagnosis at Fujian Maternity and Child Health Hospital from January 2018 to December 2019. Relative expression levels of ERRβ and ERRγ mRNA in ectopic and ectopic endometrial tissues were measured using fluorescence quantitative PCR. Serum levels of ERRβ, ERRγ, and fertility-related hormones (AMH, FSH, LH, CA125) were assessed. Correlations were analyzed, and the predictive value of ERRγ for postoperative pregnancy was evaluated using a nomogram based on LASSO and multivariate logistic regression. Internal validation using bootstrapping techniques assessed the nomograms performance, including calibration and DCA.

**Results:**

ERRβ and ERRγ mRNA levels from ectopic tissues were significantly reduced in patients with severe EMs compared to controls. High serum CA125 correlated with increased ERRγ mRNA expression in ectopic tissues. ERRγ mRNA expression in ectopic endometrial tissues was negatively correlated with age, BMI, and FSH levels, and positively with AMH and LH/FSH ratio. ERRγ mRNA and FSH were significant predictors of postoperative pregnancy, with the nomogram model showing a Brier score of 0.175 and a consistency statistic of 0.811.

**Conclusions:**

ERRβ and ERRγ are downregulated in ectopic tissues from severe EMs. Elevated ERRγ mRNA expression and lower FSH levels are predictive factors for successful postoperative pregnancy.

## 1 Introduction

Endometriosis (EMs) (1) is a chronic inflammatory disease prevalent in reproductive-age women that depends on estrogen, resulting in symptoms like dysmenorrhea, dyspareunia, and infertility, significantly affecting female reproductive health and quality of life (2). EMs results in approximately 50% of reproductive-age women facing infertility. Assisting these women in achieving successful pregnancies remains an unwavering goal of many scientists and healthcare practitioners. Patients desiring fertility can consider conservative surgery, although this may affect ovarian function. Currently, no precise model is available to predict successful postoperative pregnancy.

The estrogen receptors estrogen receptor-alpha and estrogen receptor-beta are key mediators of estrogen function and play a significant role in the onset and progression of Ems (3, 4). Estrogen-related receptors (ERRs) are a class of transcriptional regulatory factors in the nuclear receptor family that share homology with estrogen receptors. They include three subtypes: estrogen receptor-related receptor-alpha (ERRα), estrogen receptor-related receptor-beta (ERRβ), and estrogen receptor-related receptor-gamma (ERRγ) (5). The DNA-binding and ligand-binding domains of ERRs exhibit up to 68% sequence homology with ERs (6). ERRs show varying degrees of correlation in gynecologic hormone-related malignancies like breast, ovarian, and endometrial cancer and other tumors (7–9). Currently, there is no domestic or international literature on the expression and related mechanisms of ERRβ and ERRγ in severe EMs.

Approximately 30%–50% of women with EMs suffer from infertility. AMH is a marker of ovarian reserve, reflecting the remaining number of antral follicles, and its lower levels in EMs patients indicate diminished fertility potential. Compared to other benign gynecological conditions, such as ovarian cysts, women with EMs typically have lower serum levels of anti-Müllerian hormone (AMH), suggesting that EMs may impair ovarian reserve function and affect female fertility (10–12). Additionally, follicle-stimulating hormone (FSH) plays a critical role in follicular development, and elevated FSH levels may indicate impaired ovarian function (13). Luteinizing hormone (LH), essential for ovulation and the luteal phase, can also be disrupted in EMs, contributing to menstrual irregularities and infertility (14–16). Cancer antigen 125 (CA125), although not a specific marker, is often elevated in patients with Ems (17), reflecting the inflammatory nature of the disease and being used to monitor disease severity and recurrence (18–21). However, Lipari C et al. proposed that serum AMH levels in patients with EMs do not significantly change with the progression of EMs stages (22). Consequently, it is essential to identify new predictive factors to assess the likelihood of successful postoperative pregnancy in patients with severe EMs who are suspected of reduced ovarian reserve. This will enable better medical interventions and treatment guidance.

This study assessed the expression variances of ERRβ and ERRγ in endometrial tissues and serum of patients with severe EMs compared to those in the control group. Moreover, this study explored the correlation between ERRβ and ERRγ, severe EMs and fertility indicators. Evaluate the feasibility of using ERRβ and ERRγ to assist in predicting postoperative fertility in severe EMs.

## 2 Materials and methods

### 2.1 Study design

This research received approval from the Research Ethics Committee of Fujian Maternity and Child Health Hospital (Approval No. 2016-038). All participants provided written informed consent for the collection of their data. The staging and scoring of endometriosis were conducted in accordance with the “Revised American Society for Reproductive Medicine Staging System” established in 1997.

**Figrue 1** showed the flowchart of participants selection. A comprehensive description of the participant selection process is provided in the **Supplementary Materials**. In brief, the case group comprised 81 patients who were diagnosed with severe endometriosis (stages III and IV) based on postoperative histopathological analysis conducted between January 2018 and December 2019 at Fujian Maternity and Child Health Hospital. Each patient underwent laparoscopic surgery. For comparison, the control group consisted of 38 patients who also underwent laparoscopic surgery during the same period for benign gynecological conditions, such as tubal lesions or uterine scar diverticulum, and were confirmed to have normal endometrial tissue through histopathology. The criteria for inclusion and exclusion in the control group were identical to those applied to the case group. Among the 81 cases of severe endometriosis, we monitored pregnancy outcomes over a 24-month follow-up period via telephone or email, excluding instances of infertility attributable to male factors. The endpoint for the follow-up was defined by clinical pregnancy outcomes. Cases where pregnancy outcomes could not be tracked up to the final follow-up visit for any reason were classified as lost to follow-up.

**Figure 1:**
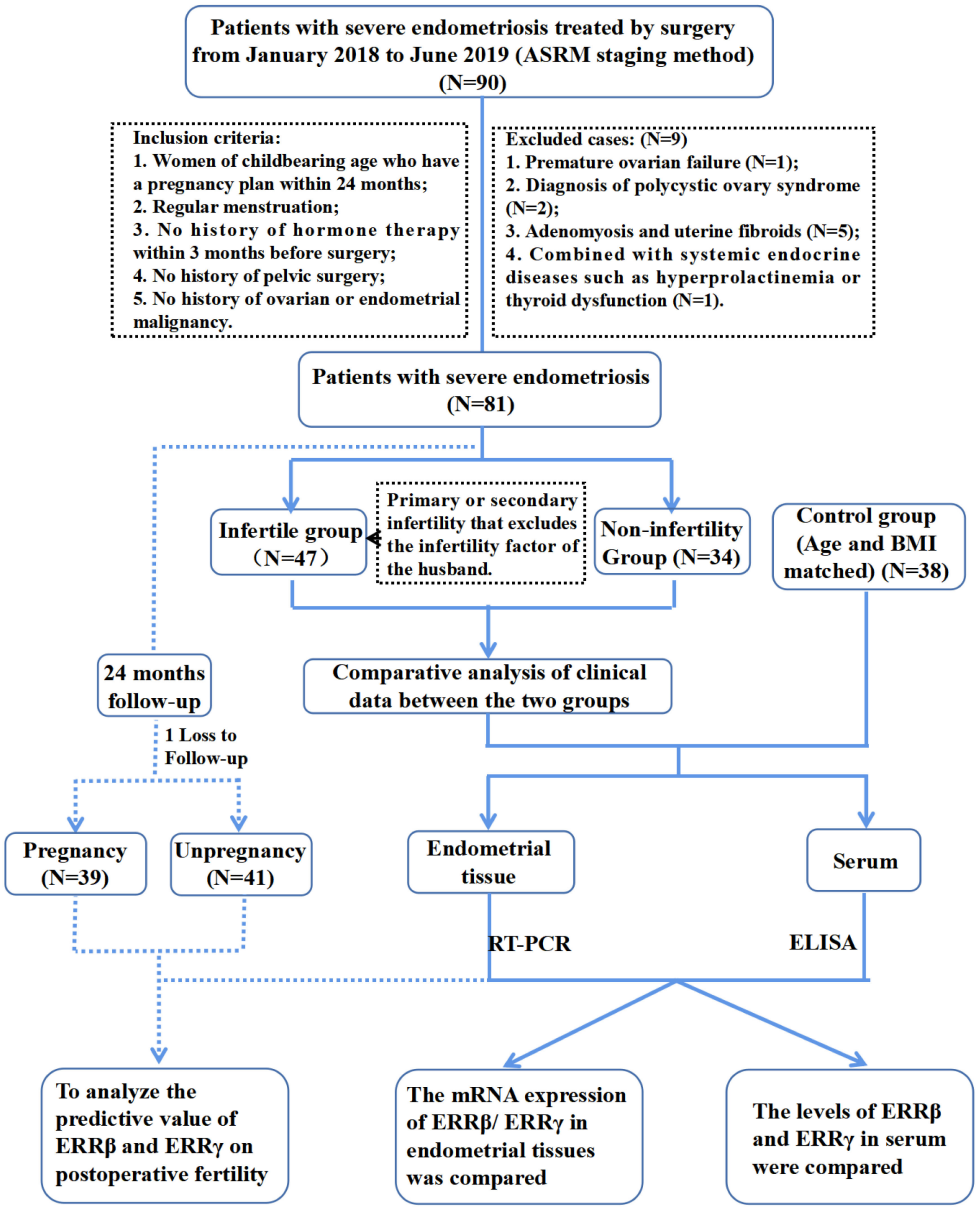
Flowchart of participants selection.

### 2.2 Real-time quantitative polymerase chain reaction

We extracted total RNA from tissue samples using the TRIZOL reagent kit (Catalog No: 15596026, Invitrogen) following the manufacturer’s instructions. We assessed RNA purity by measuring the optical density ratio at 260 nm and 280 nm, ensuring values fell between 1.9 and 2.0. We then performed reverse transcription of the extracted RNA using the High-Capacity RNA-to-cDNA kit (Catalog No: 4388950, Applied Biosystems) and stored the resulting complementary DNA at –20°C for further use.

Next, we conducted PCR on an ABI 7300 Real-Time PCR System (Applied Biosystems) using the SYBR Green PCR Master Mix (Applied Biosystems). We defined the cycle threshold (CT) as the cycle where the fluorescence signal exceeded a predetermined threshold, set between 0.1 and 0.3. We automatically calculated the CT values for the mRNA samples based on mRNA abundance using the 2–ΔΔCt method. We designed the primer sequences as follows:

ERRβ Forward: 5’-TCAAGTGCGAGTACATGCTC-3’.

ERRβ Reverse: 5’-GAAATTTGTAAGCTCAGGTA-3’.

ERRγ Forward: 5′-GCCCTCACTACACTGTGTGAC-3′.

ERRγ Reverse: 5′- CCCACCGTGTTCTTCAGACT-3′.

GAPDH Forward: 5′-GCACCGTCAAGGCTGAGAAC-3′.

GAPDH Reverse: 5′-TGGTGAAGACGCCAGTGGA-3′.

### 2.3 Quantification of serum ERRβ and ERRγ via ELISA

Following the collection of blood samples, the serum was separated by centrifugation at 1500 rpm for 10 minutes. The serum samples were then stored at –70°C until they were analyzed later that same day. To quantify the levels of ERRβ and ERRγ in the serum, a sandwich ELISA kit (Shanghai Jianglai Biotechnology Co., Ltd.) was employed. The optical density at 450 nm for each sample was measured using an ELISA reader. The concentrations of ERRβ and ERRγ in the serum were determined by referencing a standard curve (23).

### 2.4 Quantification of serum LH and FSH and BMI

Serum LH and FSH levels were performed using ELISA, as described above. Additionally, BMI was calculated as weight in kilograms divided by height in meters squared (kg/m²).

### 2.5 Development and validation of the nomogram

Univariate Cox regression analysis identified survival-associated genes, followed by LASSO (least absolute shrinkage and selection operator) regression using the R package “glmnet”. The selected predictors were incorporated into the final Cox regression model. The regression coefficients were then used to construct the nomogram. The nomogram was implemented using the R package rms, In this step, each predictor was assigned a score proportional to its contribution to the outcome, and the cumulative score across all variables provided an individualized prediction of the outcome. The model’s discriminative power was assessed by calculating the area under the curve (AUC). To assess the internal validity, we performed 1000 bootstrap resamples to estimate the optimism-corrected performance of the nomogram. The clinical applicability of the nomogram was further assessed via Decision Curve Analysis (DCA) (24).

### 2.6 Statistical analysis

For continuous variables, independent sample t-tests were utilized. Kaplan-Meier method was employed to generate survival curves, with the log-rank test applied for time-to-event analysis. A significance threshold of α = 0.05 was established, and differences were deemed statistically significant when P < 0.05.

## 3 Results

### 3.1 Expression of ERRβ and ERRγ mRNA in tissues

The baseline characteristics of the study participants are presented in **Supplementary Table 1**. There were statistically non-significant differences in the average age and body mass index (BMI) between the two groups (P > 0.05). Serum CA125 levels were significantly higher in patients with severe EMs compared to the control group (82.35 ± 85.47 versus 14.23 ± 8.04; P < 0.001), while no statistical difference was observed between the two groups in serum AMH levels (3.62 ± 3.20 versus 3.42 ± 1.86; P > 0.05).

**Figure 2:**
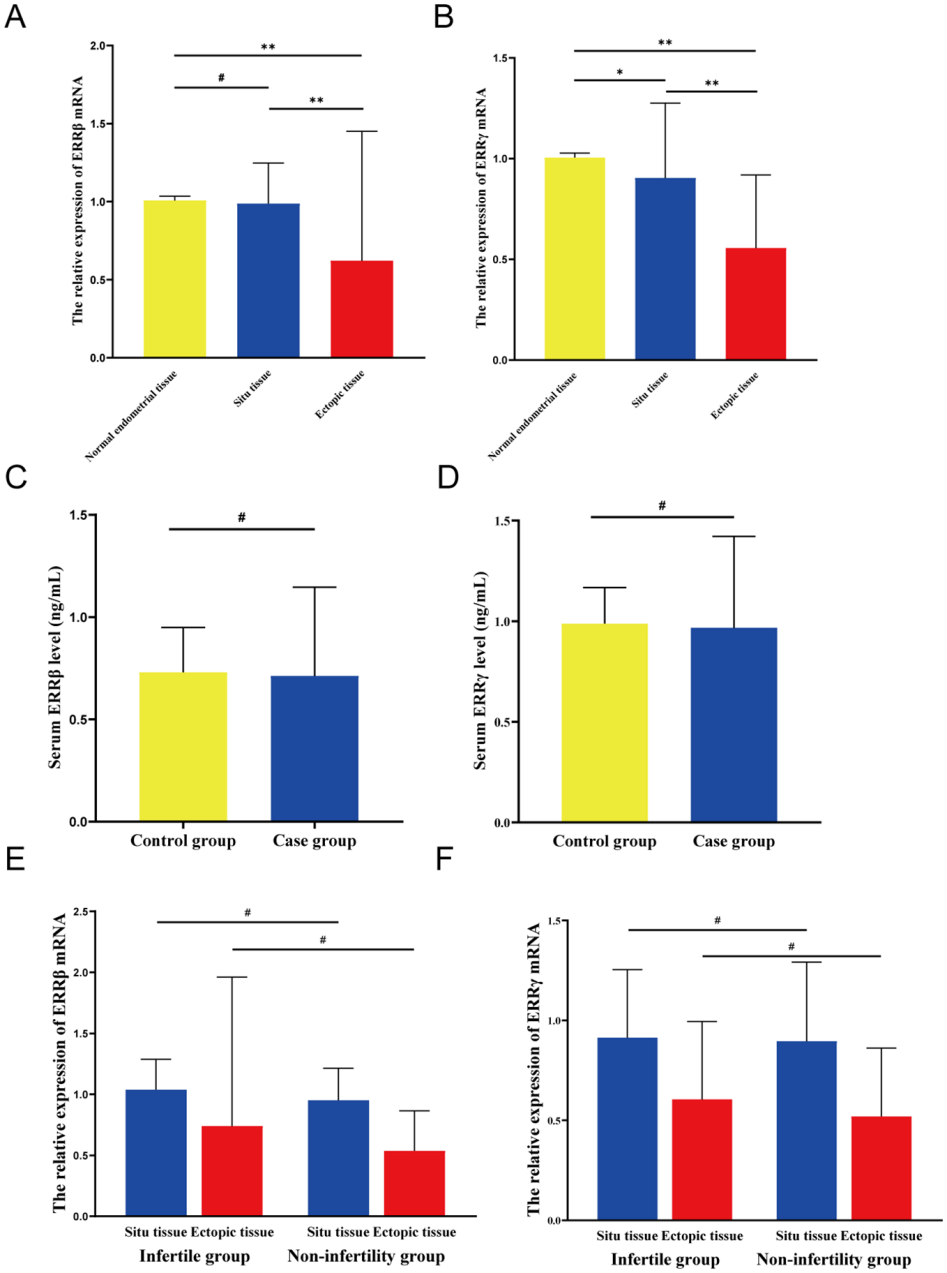
Expression levels of ERRβ/ERRγ. **(A)** Relative expression level of ERRβ mRNA. **(B)** Relative expression level of ERRγ mRNA. **(C)** Serum ERRβ expression level. **(D)** Serum ERRγ expression level. **(E)** The relative expression level of ERRβ mRNA in the groups with or without infertility. **(F)** Relative expression level of ERRγ mRNA in the groups with or without concomitant infertility. except *P < 0.05; **P < 0.01; #P > 0.05.

In patients, ectopic endometrial tissues exhibited a significant reduction in ERRβ mRNA levels compared to normal endometrial tissues from controls (P < 0.01). Conversely, no statistically significant difference was observed in ERRβ mRNA levels of *in situ* endometrial tissues between patients and controls (P > 0.05). Moreover, within the patient group, ectopic endometrial tissues showed significantly lower ERRβ mRNA levels than situ endometrial tissues (P < 0.01) (**Figure 2A**). Moreover, the relative content of ERRγ mRNA in ectopic endometrial tissue and ectopic endometrial tissue was significantly lower in the case group than in normal endometrial tissue of the control group (P < 0.01). Furthermore, the relative content of ERRγ mRNA in the ectopic endometrial tissue of the case group was significantly lower than that in the ectopic endometrial tissue (P < 0.01) (**Figure 2B**).

The serum ERRβ levels in the case group were like those in the control group, with a statistically non-significant difference (P > 0.05; **Figure 2C**). Furthermore, the serum levels of ERRγ in the case group and the control group were non-significantly different (P > 0.05; **Figure 2D**).

### 3.2 Correlation of ERRβ and ERRγ mRNA in case group with fertility-related parameters

In the case group, the expression levels of ERRβ and ERRγ mRNA in ectopic and ectopic endometrial tissues were non-significantly associated with various fertility-related parameters, including age, BMI, serum AMH, Luteinizing hormone (LH), follicle-stimulating hormone (FSH), and the LH/FSH ratio (P > 0.05, **Table 1**). The expression level of ERRγ mRNA in ectopic endometrial tissue was non-significantly correlated with fertility-related parameters in the case group (P > 0.05). Furthermore, in the case group, the relative expression level of ERRγ mRNA in ectopic endometrial tissue was significantly negatively correlated with age, BMI, and serum FSH level (r = –0.367, P = 0.001; r = –0.323, P = 0.003; r = –0.306, P = 0.008), whereas it was positively correlated with AMH and the LH/FSH ratio (r = 0.289, P = 0.009; r = 0.233, P = 0.046) (**Table 1**).

**Table 1:** Correlation between ERRβ mRNA and fertility-related indicators in the case group.

	ERRβ (Situ endometrial tissue)		ERRβ (Ectopic endometrial tissue)		ERRγ (Situ endometrial tissue)		ERRγ (Ectopic endometrial tissue)	
	r^a^	P value	r^b^	P value	r^a,^	P value	r^b,^	P value
Age (years)	-0.149	0.184	0.084	0.457	-0.367	0.001**	0.131	0.244
BMI (Kg/m²)	-0.191	0.088	-0.072	0.524	-0.323	0.003**	-0.118	0.296
AMH	0.144	0.199	-0.119	0.288	0.289	0.009**	-0.071	0.528
LH	-0.144	0.220	-0.048	0.685	-0.153	0.194	-0.011	0.924
FSH	-0.219	0.061	0.135	0.250	-0.306	0.008**	0.097	0.412
LH/FSH	0.163	0.116	-0.180	0.056	0.233	0.046*	-0.145	0.216

^a^Correlation between ERRβ mRNA and various fertility indicators in the endometrium; ^b^Correlation between ERRβ mRNA and various fertility indicators in the ectopic endometrium. *P < 0.05; **P < 0.01.

### 3.3 Relationship between Infertility and ERRβ and ERRγ mRNA expression in the case group

In the case group, patients with and without infertility showed non-significant differences in age, BMI, serum AMH level, LH/FSH ratio, estradiol (E2) level, and CA125 level (P > 0.05). Comparison between the two groups revealed significantly lower levels of serum LH and FSH in the infertility group than in the non-infertility group (P = 0.021 and P = 0.035, respectively; **Supplementary Table 2**). We compared the expression of ERRβ and ERRγ mRNA in ectopic and ectopic endometrial tissues of patients with or without infertility in the case group (**Figures 2E, F**). The results indicated that irrespective of whether the endometrial tissue was ectopic or ectopic, the infertility group exhibited higher levels of ERRβ and ERRγ mRNA than the non-infertility group, but these differences were statistically non-significant (P > 0.05).

### 3.4 Relationship between ERRβ and ERRγ mRNA and serum CA125

In a follow-up study of 81 patients with severe EMs over 24 months postoperatively, 39 patients became pregnant, 41 remained non-pregnant, and 1 patient was lost to follow-up. The postoperative pregnancy success rate in the case group was 48.8% (39/80). Patients were divided into two groups based on postoperative pregnancy outcomes in the severe EMs cohort (**Supplementary Table 3**).

**Figure 3:**
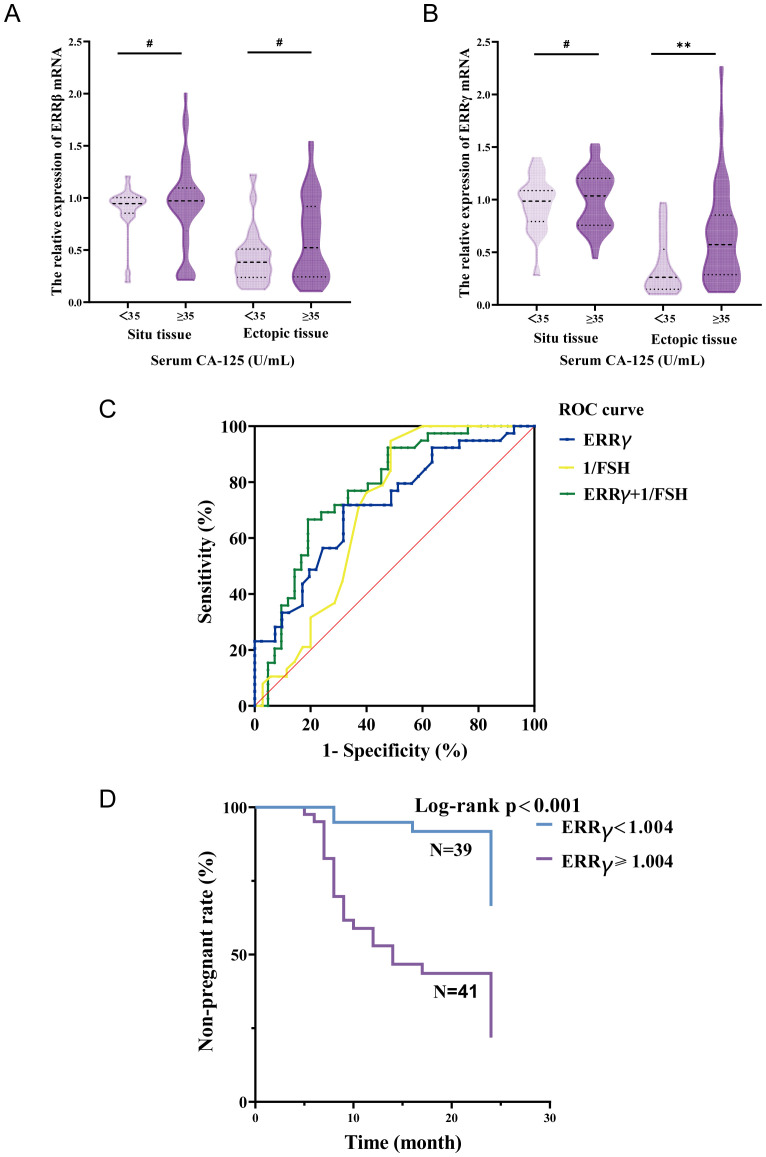
**(A)** The relationship between serum CA-125 and endometrial tissue ERRβ mRNA. **(B)** Relationship between serum CA-125 and endometrial tissue ERRγ mRNA. **(C)** ROC curve predicts postoperative pregnancy. **(D)** Non-pregnancy rate curves at 24 months after surgery in the case group. **P < 0.01; #P > 0.05.

As demonstrated in **Figure 3A**, ERRβ mRNA expression in both ectopic and *in situ* endometrial tissues showed no statistically significant differences among patients with different serum CA125 levels (P > 0.05). In contrast, patients with severe endometriosis in the high CA125 group exhibited a significant increase in ERRγ mRNA expression in ectopic endometrial tissue compared to the normal CA125 group (P < 0.01) (**Figure 3B**). However, there was still no statistically significant difference in ERRγ mRNA expression in *in situ* endometrial tissues between these groups (P > 0.05). ROC curve analysis was performed to further assess the predictive value of ectopic endometrial ERRγ mRNA expression and serum FSH levels in predicting postoperative pregnancy success in patients. The results revealed that the area under the curve (AUC) for ERRγ and 1/FSH was 0.716 and 0.699, respectively. Interestingly, when combined, the predictive value for postoperative pregnancy success increased significantly (AUC = 0.774; P < 0.001; sensitivity = 0.667; specificity = 0.805), demonstrating a more robust predictive performance (**Figure 3C**). The case group was divided into two subgroups based on the ERRγ cutoff value. One subgroup had ERRγ mRNA < 1.004 with an average age of 35.79 years and median time to pregnancy of 20.949 months. The other subgroup had ERRγ mRNA ≥1.004 with an average age of 30.62 years and median time to pregnancy of 14.098 months. Survival analysis showed a significant difference between the two groups (P < 0.001) (**Figure 3D**).

### 3.5 Predictive factors for postoperative pregnancy in patients with severe EMs

In a follow-up study of 81 patients with severe EMs over 24 months postoperatively, 39 patients became pregnant, 41 remained non-pregnant, and 1 patient was lost to follow-up. The postoperative pregnancy success rate in the case group was 48.8% (39/80).

**Figure 4:**
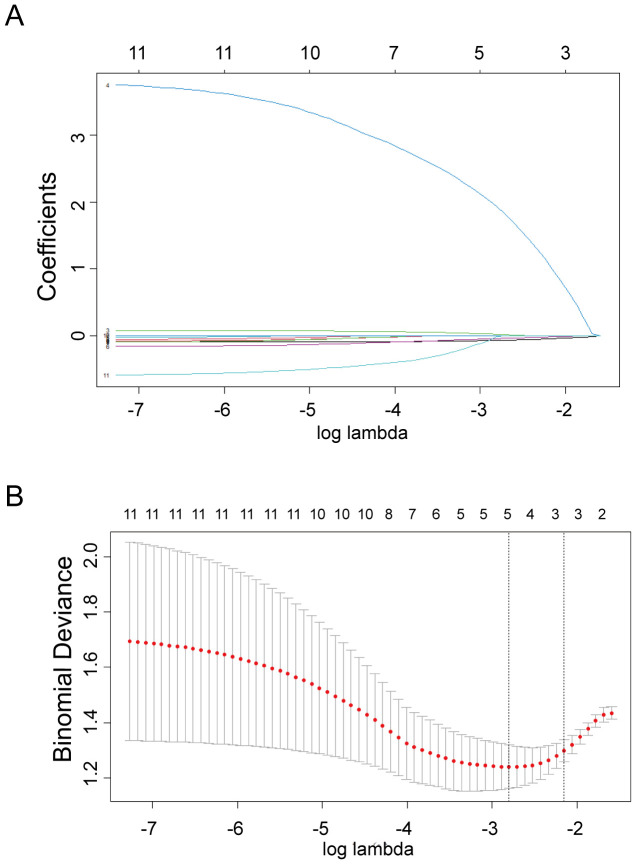
Selection of predictors through the LASSO regression analysis. **(A)** The process involved tuning parameter (lambda) selection via 10-fold crossvalidation and plotting binomial deviance against log (lambda). The dotted vertical lines were plotted at the optimal values as per the 1-SE criteria. **(B)** LASSO regression coefficient profiles of variables. A coefficient profile plot was generated against the log (lambda) sequence. Three non-zero coefficients were selected and employed to establish the prognostic model.

**Figure 5:**
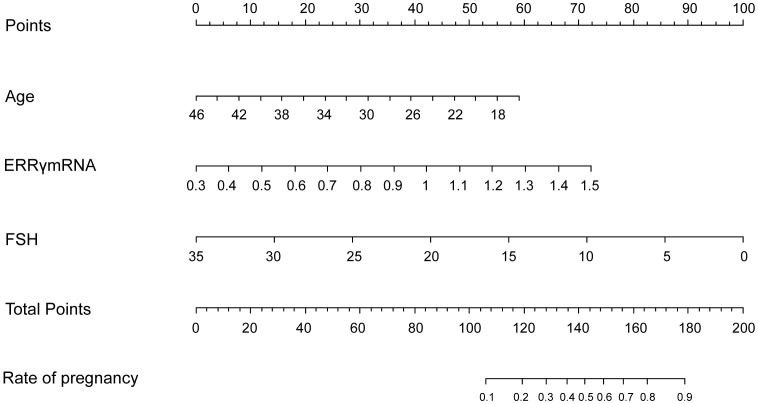
Nomogram predicts postoperative pregnancy success. The nomogram offers a means to predictive factors for postoperative pregnancy success. When using the nomogram, the points for each predictor (variable) of a patient on the uppermost rule should be located. Subsequently, the total points are obtained by adding all points. Ultimately, the corresponding predicted probability of postoperative pregnancy success on the lowest rule is identified.

**Figure 6:**
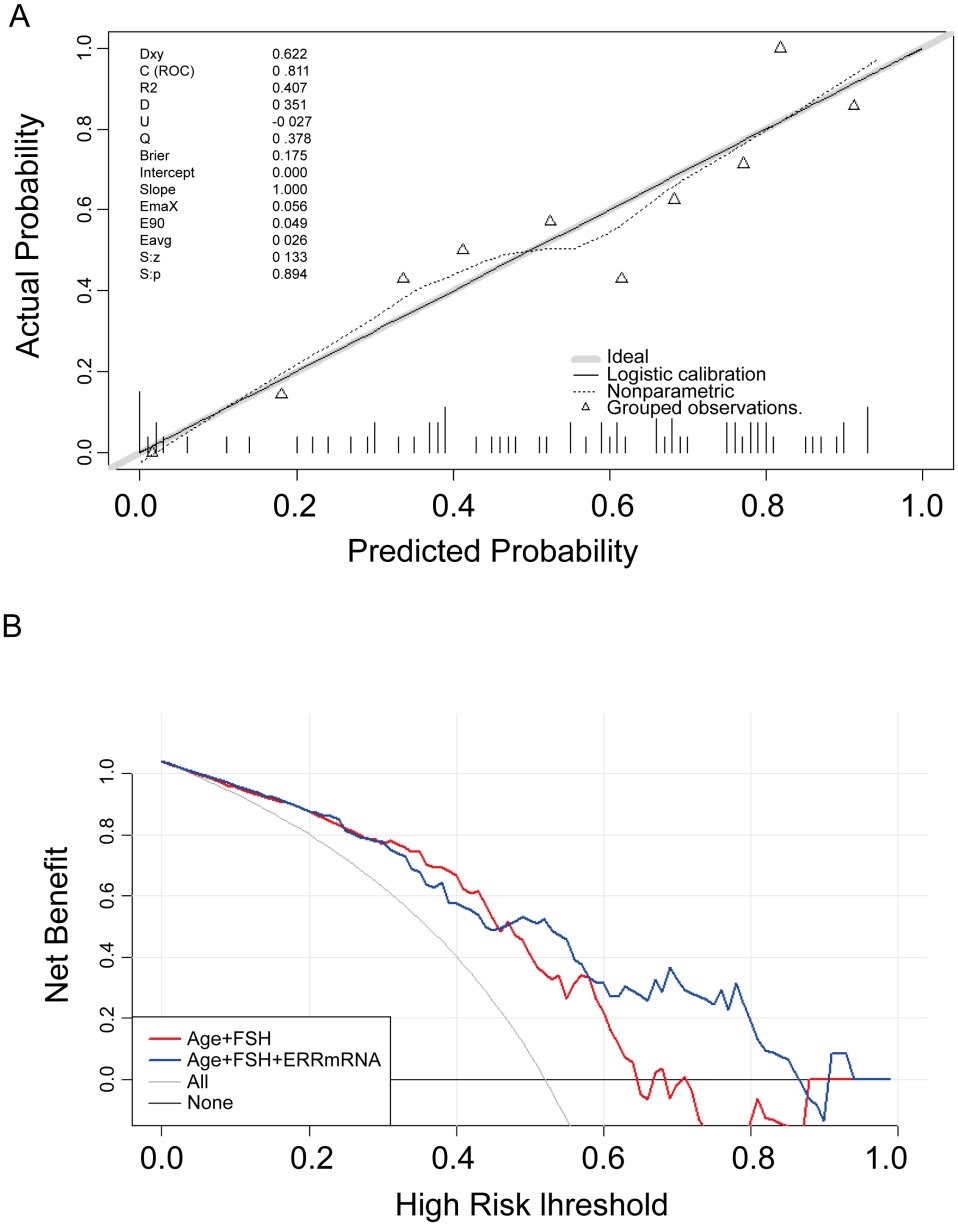
Verification of the nomogram model. **(A)** Nomogram calibration plot. The proximity of the solid line (signifying performance nomogram) to the dotted line (representing the ideal model) indicates the accuracy of the predictions of the nomogram. **(B)** Decision curve analysis of the nomogram. The red and blue solid line represents the nomogram. The graph displays the expected net benefit per patient in relation to the pregnancy success predicted by the nomogram.

LASSO regression was used to screen variables in the verification set, and finally screened the 1SE criterion, and three significant predictors of postoperative pregnancy success included in the prediction model (age, ERRγ mRNA and FSH) (**Figure 4**). Multivariate Logistic regression analysis of the selected variables showed that ERRγ mRNA (OR 36.93, 95% CI: 3.24 ~ 656.16, P = 0.007) was statistically significant (**Table 2**). As showed in **Figure 5**, age, ERRγ mRNA and FSH were included in the construction of a nomogram model to predict the probability of postoperative pregnancy success (range of 0.1 to 0.9). Clinicians can make risk predictions based on nomogram pairs to formulate individualized treatment plans. After conducting 1000 internal Bootstrap self-sampling verification on the model, the Brier score was 0.175, indicating that the model had good calibration; the consistency statistic was 0.811, indicating that the prediction model had good discrimination (**Figure 6A**). DCA evaluates the clinical application value of the predictive model. It can be seen from **Figure 6B** that at around threshold probability 0.6, the prediction model constructed using the combined indicator of age +ERRγmRNA+FSH (blue line) had higher clinical application value than age +FSH (red line) for evaluation.

**Table 2:** The logistic regression analysis of postoperative pregnancy in the case group.

	P value	OR	OR 95% CI	
			Lower	Upper
Age	0.164	0.90	0.77	1.04
AMH	0.562	1.07	0.86	1.38
ERRγ mRNA	0.007	36.93	3.24	656.16
LH	0.967	0.99	0.62	1.63
FSH	0.279	0.86	0.63	1.10

CI, confidence interval; OR, odds ratio.

## 4 Discussion

In this study, the relative expression levels of ERRβ and ERRγ mRNA in normal endometrial tissue were significantly higher in ectopic and ectopic endometrial tissues than in patients with severe EMs. However, there was a statistically non-significant difference in serum ERRβ and ERRγ levels between patients with severe EMs and the normal control group. Furthermore, ERRγ mRNA expression levels in ectopic endometrial tissue and serum FSH levels in patients with severe EMs were associated with postoperative pregnancy success. Patients with higher ERRγ expression levels and lower FSH levels had a higher success rate in achieving postoperative pregnancy.

Studies have revealed that similar to ERα and ERβ, ERRβ and ERRγ are members of the orphan nuclear receptor family. Like ERs, they can coordinate estrogen action through post-translational modifications, including phosphorylation, and exhibit significant sequence homology (25, 26). The functions of ERRs are also regulated by coactivators, including peroxisome proliferator-activated receptor γ coactivator (PGC)-1α and 1β (27). Both PGC-1α and PGC-1β are expressed in the human endometrium. In this study, ERRβ and ERRγ mRNA were relatively highly expressed in normal endometrial tissue. Cavallini et al. found that in patients with EMs, ERRγ mRNA and protein expression levels in ectopic endometrial tissue were significantly lower than those in ectopic endometrial tissue and normal endometrial tissue (28). ERRβ expression levels were similar across all three tissue types. In this study, patients with severe EMs (stages III and IV) showed a decreasing trend in the relative expression levels of ERRβ and ERRγ mRNA in both ectopic and ectopic endometrial tissues compared to normal endometrial tissue. The decrease in expression levels of both ERRβ and ERRγ was more pronounced in ectopic tissues. Additionally, ELISA assays measuring serum ERRβ and ERRγ levels revealed lower levels in patients with severe EMs compared to the normal population, although these results did not show statistical significance. This discrepancy could be attributed to the low expression levels of these receptors in the serum, potentially falling below the minimum detectable range of ELISA kits, leading to unstable experimental results.

Infertility is a major clinical manifestation of severe EMs (29, 30), yet research on characteristic biomarkers related to the impact of severe EMs on fertility outcomes remains limited. Tremblay G et al. discovered that the orphan nuclear receptor ERRβ is expressed in undifferentiated trophoblast stem cell lines and the extraembryonic endoderm, with 17β-estradiol serving as a ligand for ERRβ to regulate trophoblast stem cell differentiation (31). ERRβ expression during embryonic development defines a subset of the extraembryonic endoderm that leads to the formation of chorionic villi, suggesting an important role for ERRβ in embryonic development (32, 33). Additionally, C.E. Senner et al. found that ERRβ regulates early-stage development of the mouse trophoblast lineage, shedding light on a better understanding of human early trophoblast differentiation (34). Collectively, these studies suggest a significant role for ERRβ during early pregnancy. However, in our study, we observed a non-significant difference in ERRβ mRNA expression between patients with severe EMs, with and without infertility before surgery. Furthermore, we did not find any association between preoperative tissue ERRβ expression and postoperative pregnancy outcomes. Consequently, the relationship between ERRβ and postsurgical fertility potential in patients with severe EMs requires further exploration to understand its impact on reproductive success in this context fully.

ERRγ is highly expressed in the human placenta and significantly upregulated during human trophoblast differentiation (35). Studies have shown that microRNA may regulate trophoblast function by targeting ERRγ (36, 37). Researchers have found that ERRs play a crucial role in regulating the expression of various energy metabolism genes, influencing cellular behavior through pathways like glycolysis, lipid metabolism, and the tricarboxylic acid cycle (38–40). The results of this study imply that ERRγ may play a role in the ectopic development of endometriomas and potentially impact fertility outcomes. Additionally, correlation analysis revealed a negative association between ERRγ expression in ectopic endometrium and age, BMI, and serum FSH levels, while a positive correlation was observed with AMH levels. Higher levels of ERRγ in the ectopic endometrium are linked to better fertility potential, while lower levels may impact fertility. ERRγ could be a useful indicator of postoperative fertility in severe EMs.

In severe EMs, ectopic endometrial lesions are scattered throughout the pelvic cavity, leading to the formation of endometriotic cysts in various areas. This condition not only reduces ovarian reserve function, but surgical interventions may also affect ovarian function, thereby adversely affecting fertility (41). Anti-Müllerian hormone (AMH) is widely recognized as a marker of ovarian reserve, with several studies reporting that women with endometriosis (EMs) tend to have lower AMH levels compared to those with other benign gynecological conditions, such as ovarian cysts (10–12, 42, 43). However, in our study, we did not observe a significant difference in AMH levels between the case and control groups. This discrepancy may be explained by the sample size of our study which might have limited the statistical power to detect subtle differences between the groups. Studies on postoperative pregnancies in patients with deeply infiltrating EMs have shown a pregnancy rate of approximately 40%–42% (44). In our study of 80 patients with severe EMs, the pregnancy rate within two years postoperatively (including natural and assisted reproduction) was 48.8%. Patients experiencing infertility within two years post-surgery typically have lower preoperative levels of AMH, which aligns with previous research findings (45).

In the process of ageing in females, fertility diminishes, characterized by declining levels of AMH and rising basal Follicle Stimulating Hormone (FSH) concentrations, which coincide with diminished oocyte integrity. Such deterioration in oocyte quality is likely attributed to escalated oxidative stress and DNA damage, alongside decreased metabolic and meiotic capabilities (46). In our study, a significant association was noted between reduced preoperative FSH levels and increased likelihood of achieving pregnancy following surgery. This suggests that lower FSH levels might serve as a protective mechanism, mitigating the excessive reduction of follicular reserves and thereby preserving reproductive potential. Moreover, Zaramasina L. Clark and colleagues identified a negative association between the dosage of FSH and rates of live births (47), a phenomenon potentially linked to the activation of FSH receptors which may interfere with the quantity of oocytes in patients with endometrial ectopic conditions (48). We also found that patients with higher preoperative ERRγ mRNA levels in ectopic endometrial tissue had significantly higher success rates in achieving pregnancy within two years postoperatively than those with lower ERRγ mRNA expression levels. ERRγ showed high sensitivity and specificity in predicting postoperative pregnancy outcomes, indicating its potential predictive value for fertility in patients with severe EMs following surgery.

While this study has yielded important results, it is essential to recognize its limitations. Firstly, the study sample size was small, potentially leading to experimental biases and restricting the applicability of the findings. Secondly, there was a lack of postoperative assessment or comparison of longitudinal changes in ERRγ expression levels in ectopic endometrial tissue. Lastly, the follow-up of postoperative pregnancies may not fully account for other variables that could impact ovarian function and fertility. To ensure a thorough comprehension of the impact of surgery on fertility, it is imperative to take into consideration and acknowledge these factors. Enhancing the validity and applicability of our research findings can be achieved by overcoming these limitations through the utilization of larger sample sizes, longitudinal studies, and more comprehensive follow-up assessments.

## 5 Conclusion

In conclusion, ERRβ and ERRγ show different levels of expression in severe EM patients compared to those with normal endometrium, indicating a potential link between these genes and severe EMs’ development. ERRγ is more highly expressed in severe EMs patients without infertility, but decreases in those with infertility. Higher preoperative ERRγ expression in the ectopic endometrium may predict postoperative pregnancy success. This study provides a foundation for future investigations of the pathogenesis of severe EMs and its impact on fertility outcomes.

## Data Availability

The original contributions presented in the study are included in the article/**Supplementary Material**. Further inquiries can be directed to the corresponding author.
