# Supplementary material for: Endometrial expression of ERRβ and ERRγ: prognostic significance and clinical correlations in severe endometriosis
Source: Front Endocrinol (Lausanne). 2024 Nov 29;15:1489097. doi: 10.3389/fendo.2024.1489097 (PMC11637862; doi:10.3389/fendo.2024.1489097)
Supplement: Supplementary file 1 [file DataSheet1.docx]

Supplementary Material

1. **Study design**

The case group included 81 patients diagnosed with severe (stages III and IV) EMs based on postoperative pathological diagnosis from January 2018 to December 2019 at Fujian Maternity and Child Health Hospital. All patients underwent laparoscopic surgery. The inclusion criteria were as follows: (1) Women of childbearing age (20–40 years) with a pregnancy plan within 24 months, and (2) regular menstrual cycles with normal cycle length, menstrual duration, and flow. The exclusion criteria were as follows: (1) Premature ovarian failure; (2) confirmed diagnosis of polycystic ovary syndrome; (3) coexisting adenomyosis or uterine fibroids; (4) history of malignant ovarian or endometrial tumors; (5) systemic endocrine disorders like hyperprolactinemia or thyroid dysfunction; (6) history of hormone therapy in the three months preceding surgery; (7) history of pelvic surgery; (8) absence of informed consent; (9) unclear diagnosis. The control group comprised 38 patients who underwent laparoscopic surgery for benign gynecological conditions (such as tubal lesions and uterine scar diverticulum) during the same period, with histopathological confirmation of normal endometrial tissue. The inclusion and exclusion criteria for the control group were the same as those for the case group. For the 81 included cases of severe EMs, we evaluated pregnancy outcomes over a 24-month follow-up period through telephone or email, excluding infertility due to male factors. Clinical pregnancy outcomes were determined as the endpoint of the follow-up. Cases lost to follow-up due to the inability to track pregnancy outcomes for any reason until the last visit were considered lost to follow-up. This study was approved by the Research Ethics Committee of Fujian Maternity and Child Health Hospital (Approval No. 2016 Research Approval No. 038). All participants provided written informed consent for data collection.

All enrolled patients were required to provide complete clinical data, including age, height, weight, menstrual cycle characteristics, tumor history, surgical history, history of endocrine disorders, family history, medication history, biochemical indicators, AMH and carbohydrate antigen 125 (CA125) levels, and hormone profiles. The staging and scoring of endometrioses were based on the "Revised American Society for Reproductive Medicine Staging System" in 1997.

# 2. Supplementary Tables

Supplementary Table 1. Basic clinical data for the case and control groups.

|  | Case group  (n = 81) | Control group  (n = 38) | P value |
| --- | --- | --- | --- |
| Age (years) | 33.11±6.18 | 31.63±5.16 | 0.203 |
| Height (cm) | 161.61±4.42 | 161.08±4.73 | 0.556 |
| Weight (Kg) | 54.16±7.37 | 55.43±6.82 | 0.373 |
| BMI (Kg/m²) | 20.72±2.51 | 21.33±2.17 | 0.197 |
| CA125 (U/mL) | 82.35±85.47 | 14.23±8.04 | < 0.001** |
| AMH (ng/mL) | 3.62±3.20 | 3.42±1.86 | 0.317 |

Note: **: P < 0.01.

Supplementary Table 2. Basic clinical data of patients with and without infertility in the case group.

|  | Infertility group (n = 47) | Non-infertility group (n = 34) | P value |
| --- | --- | --- | --- |
| Age (years) | 31.97±4.35 | 33.94±7.15 | 0.129 |
| BMI (Kg/m²) | 20.22±2.14 | 21.08±2.71 | 0.128 |
| AMH (ng/mL) | 4.00±2.87 | 3.34±3.42 | 0.362 |
| LH (mIU/mL) | 4.52±1.36 | 5.72±2.87 | 0.021* |
| FSH (mIU/mL) | 6.91±2.63 | 9.60±7.38 | 0.035* |
| LH/FSH | 0.700±0.226 | 0.736±0.428 | 0.665 |
| E2 (pg/mL) | 53.32±55.15 | 50.12±30.92 | 0.754 |
| CA-125 (U/mL) | 76.39±57.82 | 86.66±101.30 | 0.597 |

Note: *: P < 0.05.

Supplementary Table 3. Comparison of clinical data between the postoperative pregnancy and non-pregnancy groups.

|  | Pregnant group (n = 39) | Non-pregnant group (n = 41) | P value |
| --- | --- | --- | --- |
| Age (years) | 30.13±3.80 | 35.66±6.61 | < 0.001** |
| BMI (Kg/m^2^) | 20.25±2.09 | 21.21±2.81 | 0.088 |
| AMH (ng/mL) | 4.88±3.60 | 2.48±2.25 | 0.015* |
| LH (mIU/mL) | 4.50±1.61 | 5.72±2.68 | 0.024* |
| FSH (mIU/mL) | 6.07±1.56 | 10.53±7.44 | 0.001** |
| LH/FSH | 0.761±0.417 | 0.576±0.342 | 0.034* |
| CA125 (U/mL) | 84.92±62.60 | 81.25±103.94 | 0.850 |
| ERRγ mRNA (Situ endometrial tissue) | 1.01±0.24 | 0.81±0.24 | < 0.001** |

Note: *: P < 0.05; **: P < 0.01.
